# Development of a LAMP method with lateral flow DNA chromatography to diagnose toxoplasmosis in immunocompromised patients

**DOI:** 10.1186/s41182-024-00613-4

**Published:** 2024-07-08

**Authors:** Kei Mikita, Takehiko Mori, Tamayo Komine, Seiki Kobayashi, Satoshi Iwata, Koichi Suzuki, Naoki Hasegawa

**Affiliations:** 1 Department of Infectious Diseases, Keio University School of Medicine, 35 Shinanomachi, Shinjuku-Ku, Tokyo, 160-8582 Japan; 2 Department of Hematology, Graduate School of Medical and Dental Sciences, Tokyo Medical and Dental University (TMDU), Tokyo, Japan; 3 Department of Parasitology, National Institute of Infectious Diseases, Tokyo, Japan; 4 Department of Microbiology, Tokyo Medical University, Tokyo, Japan; 5 Department of Clinical Laboratory Science, Faculty of Medical Technology, Teikyo University, Tokyo, Japan

**Keywords:** Loop-mediated isothermal amplification, DNA chromatography, Toxoplasmosis, Toxoplasma encephalitis, *Toxoplasma gondii*

## Abstract

**Background:**

Rapid and accurate diagnosis of toxoplasmosis is critical, particularly for immunocompromised patients. Several molecular methods could have value for toxoplasmosis diagnosis, but often require sophisticated and expensive equipment, and as such are impractical for use in resource-limited countries. Our study aimed to develop a new rapid diagnostic test for toxoplasmosis that can be used in developed countries as well as low- or middle-income countries.

**Methods:**

Common primers for conventional loop-mediated isothermal amplification (LAMP) and the new LAMP DNA chromatography method were designed based on a 529-bp repeat present in *Toxoplasma gondii* genomic DNA. A total of 91 clinical samples from 44 patients suspected of having toxoplasmosis who were treated at several hospitals across Japan were tested using the new LAMP DNA chromatography method, conventional LAMP, and nested PCR and the sensitivity and specificity of the methods was compared.

**Results:**

The LAMP DNA chromatography method showed better sensitivity and specificity (68.2% and 100%, respectively) compared with the nested PCR (45.4% and 100%, respectively) and conventional LAMP (63.6% and 100%, respectively) methods for diagnosis of toxoplasmosis in immunocompromised patients. LAMP DNA chromatography also has better sensitivity and specificity (75% and 100%, respectively) than nested PCR (50.0% and 93.5%, respectively) and conventional LAMP (62.5% and 100%, respectively) to diagnose toxoplasma encephalitis using CSF samples.

**Conclusion:**

We developed a LAMP DNA chromatography method to detect *T. gondii* DNA in clinical samples. This method also successfully detected *T. gondii* DNA in CSF from patients with toxoplasma encephalitis. This newly developed method can be a valuable rapid diagnostic test for toxoplasmosis in a range of settings, including resource-limited areas like those in low- or middle-income countries.

## Introduction

*Toxoplasma gondii* is an obligate intracellular protozoan parasite that can infect both humans and any warm-blooded animal worldwide. The clinical manifestations of *T. gondii* infections are usually benign and self-limited, but can be life-threatening in congenitally infected and immunocompromised patients. In particular, reactivation of latent infection in immunocompromised individuals can cause fatal toxoplasma encephalitis, myocarditis, and pneumonitis [1]. Acute toxoplasmosis in immunocompromised patients can be rapidly lethal with estimated mortality rates ranging between 60 and 90% if treatment is not begun soon after infection [2, 3]. Therefore, effective, rapid, and accurate diagnosis is crucial to initiate appropriate treatment to achieve a good prognosis [4].

Several DNA-based detection methods including regular PCR, nested PCR, and real-time quantitative PCR have been developed to detect *T. gondii* infection [5, 6]. However, these tools require sophisticated and expensive equipment, as well as specialized training for staff who carry out these techniques. These limitations have hampered the adoption of these methods by laboratories that have limited experience in molecular testing, particularly in resource-limited countries where the incidence of *T. gondii* infection can be high.

Loop-mediated isothermal amplification (LAMP) is a gene amplification method technique that requires no expensive equipment (e.g., thermocycler) since DNA amplification occurs at a constant temperature [7]. Several reports showed that LAMP offers superior sensitivity to conventional PCR methods [8, 9]. The simplicity, rapidity, and sensitivity of LAMP, which involves only low-cost equipment, makes this approach suitable for use in resource-limited areas.

In general, LAMP products are analyzed using a fluorescent dye specific for double-stranded DNA or by measuring the turbidity of magnesium pyrophosphate formed as a by-product during the LAMP reaction. However, there is potential for misinterpretation of the fluorescent color and turbidity that would produce differing outcomes based on the same result. Although real-time turbidimeters have recently become widely available for use with the LAMP method and have shown reliable results, these devices are still expensive and thus impractical in resource-limited countries [10].

Specific DNA sequences can be detected using lateral flow dipsticks in which DNA–DNA hybridization between single-strand tag sequences attached to the 5ʹ end of primers and complementary probes adhered to the dipstick occurs without denaturation of amplicons. LAMP products labeled with blue beads and the 5ʹ single-strand tag sequence are trapped by oligonucleotides carrying a sequence complementary to the tag sequence and printed on a strip membrane to allow visualization. The reaction can be visualized by monitoring the accumulation of colorant [11, 12]. Recently, a single-stranded tag hybridization chromatographic printed-array strip (STH-PAS) genotyping method was developed [16]. Targets are amplified using primer pairs having a unique single-stranded sequence-tag and biotin labeling and developed on printed-array strips with streptavidin-coated blue latex beads that react with the biotin label [17]. This approach has been applied for visualization after LAMP amplification of *Plasmodium* spp. and *Rickettsia* spp. from human blood [18].

In this study, we developed a LAMP assay combined with DNA chromatography to detect *T. gondii* DNA. We evaluated this method using clinical samples from patients who were suspected of having toxoplasmosis.

## Material and methods

### Parasite preparation and DNA extraction

*T. gondii* RH strain tachyzoites were obtained by in vitro culture as described previously [13]. Harvested tachyzoites were first suspended in phosphate-buffered saline and counted in a counting chamber under a light microscope. The parasites in the suspension were then lysed using proteinase K. Genomic DNA was extracted from lysates using a QIAamp DNA Mini Kit (QIAGEN, Hilden, Germany) according to the manufacturer's instructions. Genomic DNA from quantified amounts of *T. gondii* tachyzoites was used as a standard to evaluate the detection limits of the nested PCR method, conventional LAMP method, and LAMP DNA chromatography method.

### Clinical samples and DNA extraction

Between 2017 and 2024, 91 clinical samples (43 blood samples, 39 cerebrospinal fluid (CSF) samples, three bronchoalveolar fluid samples, two urine samples, two brain tissue sample, one lung tissue sample, and one lymph node sample) were collected for diagnostic purposes from 44 patients who were suspected of having toxoplasma encephalitis, disseminated toxoplasmosis, congenital toxoplasmosis, or toxoplasma pneumonia based on clinical course. Imaging findings were collected at several hospitals in Japan and sent to our laboratory for genetic diagnosis. The underlying diseases of patients (excluding cases with suspected congenital toxoplasma) were acute myeloid leukemia (*n =* 11), AIDS (*n =* 7), malignant lymphoma (*n =* 4), acute lymphocytic leukemia (*n =* 4), chronic myelogenous leukemia (*n =* 3), myelodysplastic syndromes (*n =* 3), adult T-cell leukemia (*n =* 2), cancer-bearing (*n =* 2), rheumatoid arthritis (*n =* 1), marginal zone lymphoma (*n =* 1), angioimmunoblastic T-cell lymphoma (*n =* 1), aplastic anemia (*n =* 1), systemic lupus erythematosus (*n =* 1), and IgA vasculitis nephritis (*n =* 1).

Among these patients, 14 cases were finally diagnosed as true toxoplasmosis, including toxoplasma encephalitis (*n =* 10), toxoplasma pneumonia (*n =* 1), and disseminated toxoplasmosis (*n =* 3). The final diagnosis as true toxoplasmosis was made by attending physicians at each hospital based on the effectiveness of *T. gondii*-specific chemotherapy, pathological diagnosis by biopsy, or autopsy. All clinical samples were sent to our laboratory as fresh or frozen condition. Genomic DNA was extracted from the 91 clinical samples using a QIAamp DNA Mini Kit (QIAGEN) according to the manufacturer's instructions.

The design and protocol of the current study conformed to the Helsinki Declaration and were approved by the Institutional Ethics Committee. This study was appraised and approved by the Ethics Committees of Keio University School of Medicine, Tokyo, Japan (No: 20190154 and 20,200,217). Written informed consent was obtained from all subjects involved in the study.

### LAMP primer design

The LAMP primer set for detecting *T. gondii* DNA was designed to target a 529-bp repeat in *T. gondii* genomic DNA (GenBank Accession No. AF146527). The primers were designed using Primer Explorer V5 software (http://primerexplorer.jp/v5_manual/index.html; Eiken Chemical, Tokyo, Japan). Out of the six candidate primer sets comprising FIP/BIP/F3/B3, the primer set that had the fastest amplification time using a serially diluted *T. gondii* DNA template was selected and then LF and LB primers were further designed. The six primers used for the conventional LAMP method (FIP, BIP, F3, B3, LF, and LB) are shown in Table 1.

**Table 1:** LAMP primers to detect 529 bp *Toxoplasma gondii* sequence

Primer	Sequence (5ʹ → 3ʹ)
Toxo_529bp_FIP-tag^a^	GGATCGCATTCCGGTGTCTCTTAAGATGTTTCCGGCTTGGC
Toxo_529bp_BIP-biotin^b^	TCGTGGTGATGGCGGAGAGAATCCTCCTCCCTTCGTCCAA
Toxo_529bp_FIP	GGATCGCATTCCGGTGTCTCTTAAGATGTTTCCGGCTTGGC
Toxo_529bp_BIP	TCGTGGTGATGGCGGAGAGAATCCTCCTCCCTTCGTCCAA
Toxo_529bp_F3	TGGGAAGCGACGAGAGTC
Toxo_529bp_B3	TGGATTCCTCTCCTACCCCT
Toxo_529bp_LF	AAGAGTGGAGAAGAGGGCG
Toxo_529bp_LB	TCCACCCTCCAGGAAAAGC

^a^Tag sequence added to the 5ʹ end of each primer

^b^Biotin added to the 5ʹ end of each primer

**Fig. 1:**
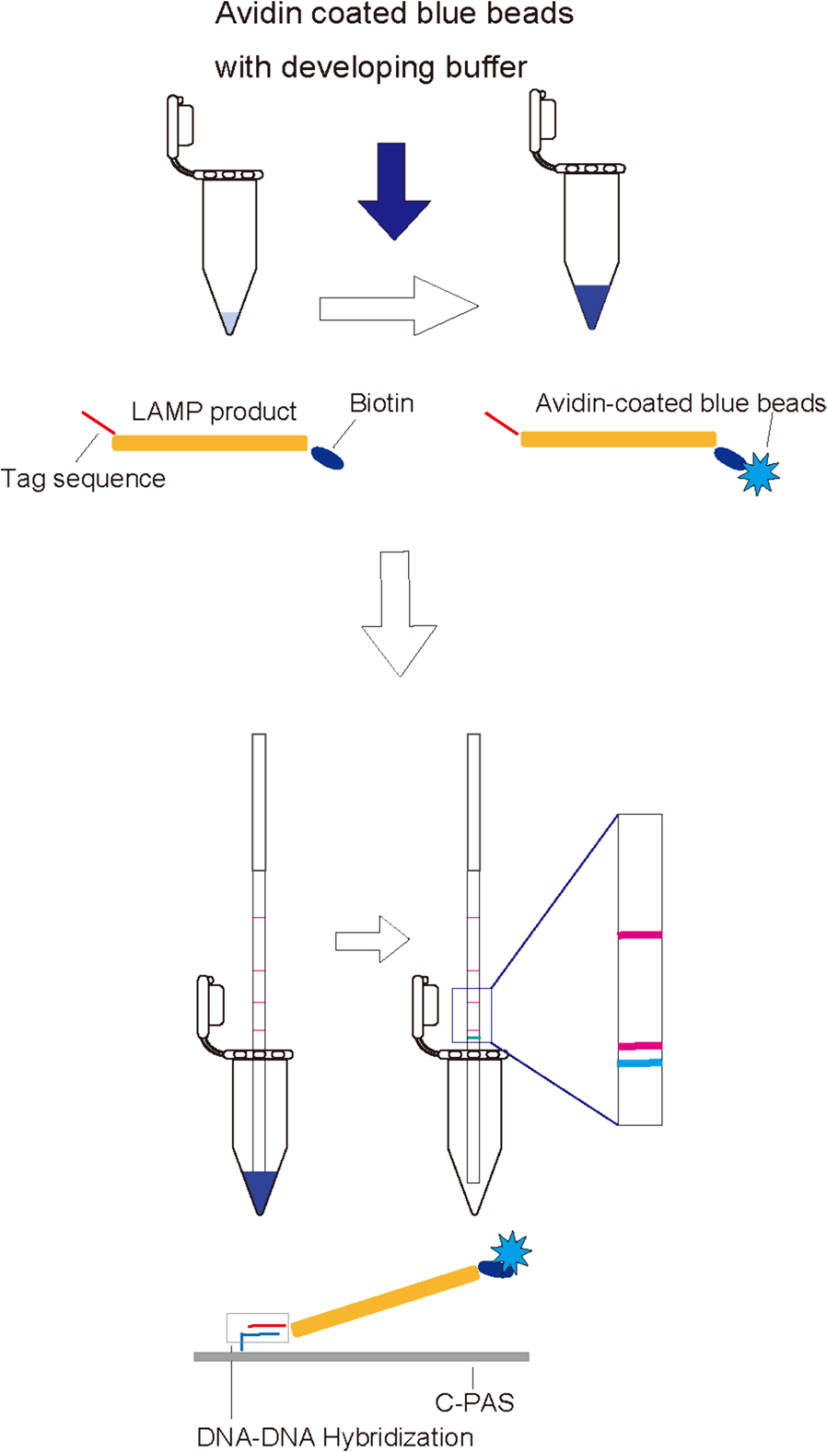
Schematic representation of LAMP DNA chromatography to detect a 529-bp *T. gondii *DNA sequence. LAMP products are labeled with a tagged sequence and biotin after amplification. The biotin-labeled LAMP products are then labeled with avidin-coated blue beads. The LAMP products labeled with the blue beads are trapped by oligonucleotides having a complementary sequence to the tag sequence at the end of the LAMP products and that are printed on a DNA chromatography strip (blue line)

We used the STH-PAS system to develop a method to detect the 529-bp fragment of *T. gondii* DNA (Fig. 1). DNA chromatography strips and reagents were obtained commercially (TBA, Sendai, Japan). For LAMP DNA chromatography, the FIP primer was labeled with a F1-tag sequence, and the BIP primer was labeled with biotin (obtained from TBA). Primers used for LAMP DNA chromatography (FIP-tag, BIP-biotin, F3, B3, LF, and LB) are shown in Table 1.

### Conventional LAMP method

The LAMP reaction was performed using a Loopamp^®^ DNA amplification kit D (Eiken Chemical, Tokyo, Japan) following the manufacturer’s instructions. The LAMP reaction was performed in a total volume of 25 μL, comprising1 μL target DNA template, 1.6 μM FIP and BIP primers, 0.2 μM of each outer primer (F3 and B3), and 0.8 μM of each loop primer (LF and LB). The reaction was performed using the real-time turbidity-measuring device Loopamp EXIA (Eiken Chemical) at 63 °C for 40 min and then for 5 min at 80 °C to inactivate the enzyme. Positive results for the LAMP reaction are based on turbidity and were determined automatically using the Loopamp EXIA.

### Development of the LAMP DNA chromatography detection method

We used the STH-PAS system to develop a detection method that will detect a 529-bp repeat fragment of genomic *T. gondii* DNA*.* Dipstick DNA chromatography strips and reagents were obtained commercially (TBA). For LAMP DNA chromatography, the FIP primer was labeled with a F1-tag sequence, and the BIP primer was labeled with biotin (purchased from TBA). The LAMP amplification proceeded for 40 min in a 63 °C heat block to generate LAMP products with a 5ʹ tag sequence and 3ʹ biotin labeling. A DNA chromatography strip (TBA) was inserted into a 21 µL reaction mixture containing 10 µL of developing solution (TBA), 9 µL distilled water, 1 µL of LAMP product, and 1 µL of avidin-coated blue beads. LAMP products labeled with the beads are trapped by oligonucleotides having a sequence that is complementary to the tag sequence printed on the strip resulting in a blue line (Fig. 1).

### Limit of detection

We determined the detection limit of the LAMP DNA chromatography method, conventional LAMP method and the nested PCR method that we previously reported [3] using tenfold serial dilutions of genomic DNA.

## Results

### Detection limit

**Fig. 2:**
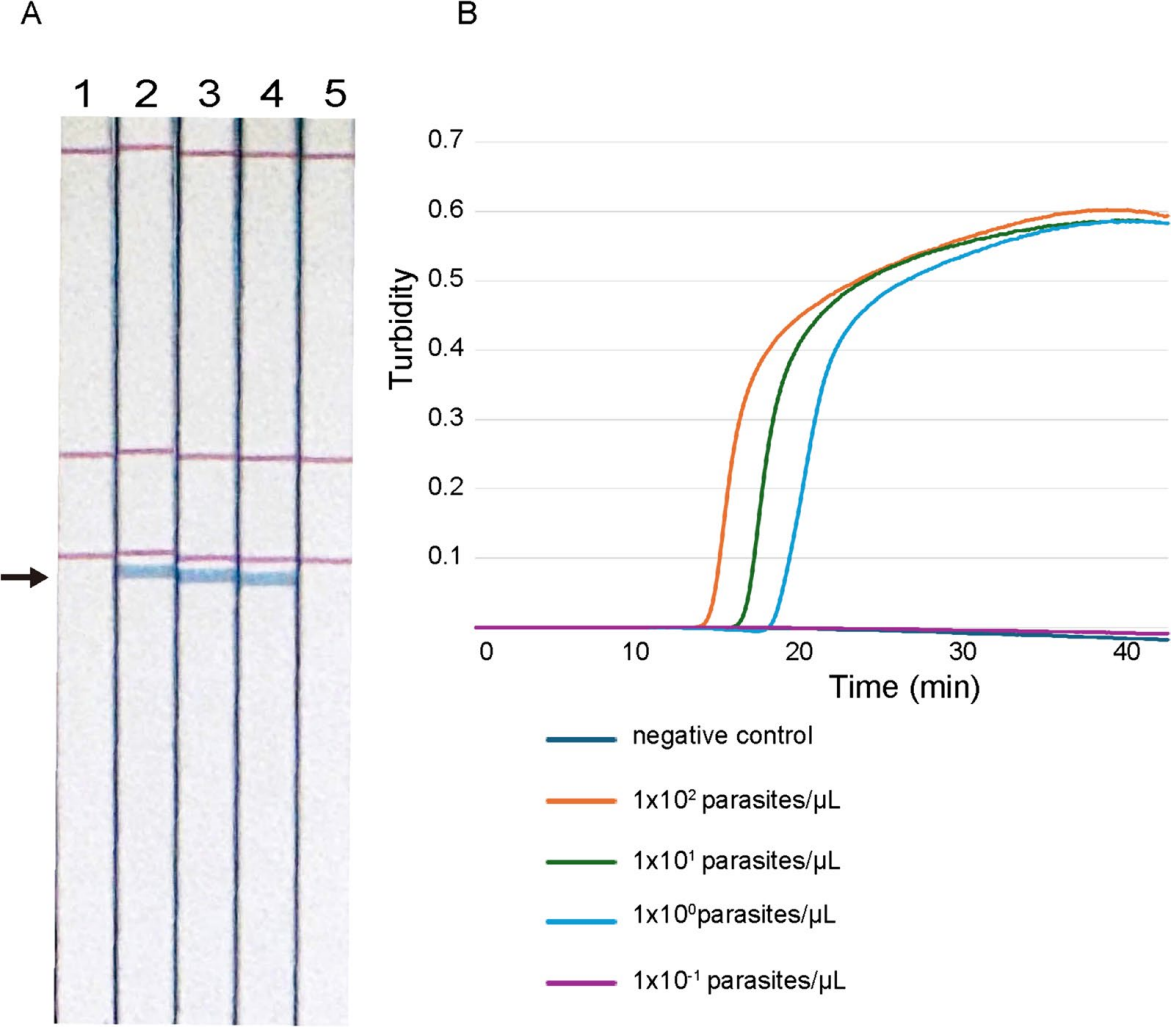
Detection limit of LAMP DNA chromatography and conventional LAMP methods. **A** Detection limit of the LAMP DNA chromatography method determined using tenfold serial dilutions of *T. gondii* genomic DNA (1: negative control, 2: 1 × 10^2^ parasites/μL, 3: 1 × 10^1^ parasites/μL 4: 1 × 10^0^ parasites/μL 5: 1 × 10^−1^ parasites/μL). **B** Detection limit of the conventional LAMP method to detect a 529-bp repeat in *T. gondii* genomic DNA was determined with tenfold serial dilutions of *T. gondii* genomic DNA

We first determined the detection limit of the nested PCR method that we previously reported, the conventional LAMP method and the LAMP DNA chromatography method with serial dilutions of genomic DNA of *T. gondii* using concentrations of parasites that ranged from 10^2^ parasites/μL to 10^–1^ parasites/μL. All three methods allowed detection of *T. gondii* genomic DNA at a concentration of 1 parasite/μL **(**Fig. 2**)**. Thus, the conventional LAMP method and LAMP DNA chromatography had a high detection limit that was comparable to that measured for nested PCR.

### Sensitivity and specificity of the conventional LAMP method, LAMP DNA chromatography, and nested PCR method

We next evaluated the sensitivity and specificity of the nested PCR method, conventional LAMP, and LAMP DNA chromatography using 91 clinical samples from 44 patients suspected of having toxoplasmosis.

The LAMP DNA chromatography method showed superior sensitivity and specificity (68.2% and 100%, respectively) for identifying toxoplasmosis using 91 clinical samples, 22 samples from 14 true toxoplasmosis cases and 69 samples from other disease cases, compared to the sensitivity and specificity for nested PCR (45.4% and 97.1%, respectively) and conventional LAMP (63.6% and 100%, respectively) methods (Table 2). Furthermore, the LAMP DNA chromatography method exhibited superior sensitivity and specificity to diagnose toxoplasma encephalitis using 39 CSF samples, 8 from toxoplasma encephalitis cases and 31 from other diseases cases (75.0% and 100%, respectively) relative to that for nested PCR (50.0% and 93.5%, respectively) and conventional LAMP (62.5% and 100%, respectively) (Table 3). The LAMP DNA chromatography method also showed 100% and 33% positive results using blood samples from disseminated toxoplasmosis and toxoplasma encephalitis, 100% brain tissue and 100% lung tissue, respectively.

**Table 2:** Conventional LAMP and LAMP DNA chromatography sensitivity and specificity

Clinical diagnosis	Nested PCR		Conventional LAMP		LAMP DNA chromatography	
	Positive	Negative	Positive	Negative	Positive	Negative
Toxoplasmosis (*n* = 22)	10 (45.4%)	12 (54.5%)	14 (63.6%)	8 (36.4%)	15 (68.2%)	7 (31.8%)
Other diseases (*n* = 69)	2 (2.9%)	67 (97.1%)	0 (0%)	69 (100%)	0 (0%)	69 (100%)

**Table 3:** Sensitivity and specificity of conventional LAMP and LAMP DNA chromatography for toxoplasma encephalitis

Clinical diagnosis	Nested PCR		Conventional LAMP		LAMP DNA chromatography	
	Positive	Negative	Positive	Negative	Positive	Negative
Toxoplasma encephalitis (*n* = 8)^a^	4 (50.0%)	4 (50.0%)	5 (62.5%)	3 (36.4%)	6 (75.0%)	2 (25.0%)
Other diseases (*n* = 31)	2 (6.5%)	29 (93.5%)	0 (0%)	31 (100%)	0 (0%)	31 (100%)

^a^CSF samples were obtained for eight of ten toxoplasma encephalitis cases

Lumbar puncture was contraindicated in two cases

## Discussion

In this study, we developed a LAMP DNA chromatography method to identify a 529-bp fragment of *T. gondii* genomic DNA*.* This method is rapid and sensitive and requires no expensive equipment or specialized technical expertise. We compared the performance of this new method with a nested PCR method we previously described and conventional LAMP. All three methods had similarly higher detection limits. However, the LAMP DNA chromatography method could diagnose toxoplasmosis in clinical samples with higher sensitivity and specificity (68.2% and 100%, respectively) than either nested PCR (45.4% and 100%, respectively) or conventional LAMP (63.6% and 100%, respectively).

Various molecular techniques for detecting *T. gondii* in clinical samples represented a significant advance in the diagnosis of toxoplasmosis [14, 15]. In immunocompromised patients, acute toxoplasmosis, including toxoplasma encephalitis and disseminated toxoplasmosis, is fatal. Therefore effective, rapid, and accurate diagnosis in which *T. gondii* DNA is detected in clinical samples is urgent to guide treatment decisions to increase the likelihood of achieving a good prognosis [2, 16]. The robust and specific performance of LAMP-based methods to detect *T. gondii* has been previously demonstrated, largely in veterinary studies. A limited number of studies described the use of LAMP-based methods with human clinical samples to diagnose toxoplasmosis [6, 9, 17, 18]. Although some LAMP methods were reported to have higher sensitivity compared with nested PCR for the detection of *T. gondii* DNA in human blood samples [9, 17], our report is the first, to our knowledge, to demonstrate detection of *T. gondii* DNA in CSF samples from patients with toxoplasma encephalitis using either conventional LAMP or a LAMP DNA chromatography method.

For diagnosis of toxoplasma encephalitis, the detection of *T. gondii* DNA in CSF using PCR is specific, but not sensitive, meaning that a positive test confirms the diagnosis, but a negative test cannot rule out presence of infection [19, 20]. In fact, the sensitivity and specificity of PCR using CSF was reported to range between 17–65% and 76.5–100%, respectively [21]. The LAMP DNA chromatography method using blood samples showed lower sensitivity than CSF samples for diagnosing toxoplasma encephalitis, 33% and 75%, respectively. Therefore, it is recommended that CSF samples be used for LAMP chromatography whenever possible in cases of toxoplasma encephalitis. In the present study, the LAMP DNA chromatography and conventional LAMP methods showed superior sensitivity and specificity compared to the nested PCR method (68.2% and 100% vs. 63.6% and 100% and 45.4% and 97.1%).

Real-time turbidimeters for use with a conventional LAMP method offer high sensitivity, but these instruments are costly. In our newly developed LAMP DNA chromatography method, the LAMP target sequence is modified with a tag sequence that allows simple identification of LAMP amplicons based on positions of blue lines on a DNA chromatography strip. The amplicons can be generated without expensive instruments like a thermal cycler and the lines on the strip are easily visualized so equipment like a UV transilluminator is not needed. The LAMP DNA chromatography method is suitable for resource-limited countries since only a heating block is needed for amplification. Furthermore, as mentioned above, the LAMP DNA chromatography sensitivity and specificity is superior to that for conventional LAMP for detection of *T. gondii*.

It is important to note that the tube containing the LAMP reaction must not be opened after amplification to prevent the spread of LAMP products. Moreover, the LAMP DNA chromatography method should be performed in dedicated, separate spaces and rooms [13]. In this respect, we are currently developing a novel, all-in-one diagnostic kit in which the LAMP method and DNA chromatography can be carried out in a completely sealed device.

## Conclusions

We developed LAMP DNA chromatography for identifying *T. gondii* DNA in clinical samples. We successfully detected *T. gondii* DNA in a range of clinical samples from patients with toxoplasma encephalitis, including CSF. Our results indicated that the LAMP DNA chromatography method could represent an ideal point of care test that can be made widely available worldwide for use in developed countries as well as low- and middle-income countries.

## Data Availability

All datasets supporting the conclusions of this study are included in the article.

## Abbreviation

LAMP: Loop-mediated isothermal amplification
